# The role of lutein-rich purple sweet potato leaf extract on the amelioration of diabetic retinopathy in streptozotocin-induced Sprague–Dawley rats

**DOI:** 10.3389/fphar.2023.1175907

**Published:** 2023-05-18

**Authors:** Ahmad Safiyyu'd-din Bin Hisamuddin, Ruth Naomi, Khairul Aiman Bin Manan, Hasnah Bahari, Fezah Othman, Hashim Embong, Amin Ismail, Qamar Uddin Ahmed, Siti Hadizah Jumidil, Mohd Khairi Hussain, Zainul Amiruddin Zakaria

**Affiliations:** ^1^ Borneo Research on Algesia, Inflammation and Neurodegeneration (BRAIN) Group, Faculty of Medicine and Health Sciences, Sabah Universiti Malaysia, Kota Kinabalu, Sabah, Malaysia; ^2^ Department of Biomedical Sciences, Faculty of Medicine and Health Sciences, Universiti Putra Malaysia, Serdang, Malaysia; ^3^ Department of Human Anatomy, Faculty of Medicine and Health Sciences, Universiti Putra Malaysia, Serdang, Malaysia; ^4^ Department of Emergency Medicine, Faculty of Medicine, Universiti Kebangsaan Malaysia, Kuala Lumpur, Malaysia; ^5^ Department of Nutrition, Faculty of Medicine and Health Sciences, Universiti Putra Malaysia, Serdang, Malaysia; ^6^ Department of Pharmaceutical Chemistry, Kulliyah of Pharmacy, International Islamic University Malaysia, Kuantan, Pahang, Malaysia

**Keywords:** purple sweet potato, plant, diabetes, cataract, retinal changes, antioxidants

## Abstract

The objective of this study is to access the effect of purple sweet potato leaf (PSPL) extract on diabetic retinopathy (DR) of streptozotocin (STZ)-induced male Sprague–Dawley (SD) rats. In this study, rats were injected intraperitoneally with a single dose of 60 mg/kg STZ, and diabetes was confirmed on day 7. Rats were further divided into a few groups, which were then orally administered with one of the following treatments: 25 mg/kg of gliclazide (D25G), 200 mg/kg of PSPL extract (DT 200), and 400 mg/kg of PSPL extract (DT 400). However, the normal control (NS) and control group for diabetic (DNS) were given normal saline (NS) for 12 weeks. The results show that the treated group demonstrated a reduction in serum oral glucose tolerance test (OGTT) levels of DT 200 and DT 400, and an increase in the serum and retinal insulin levels, and restored oxidative stress markers in serum and retina on week 12. The PSPL extract exhibited protective effects in maintaining the kidney, liver, retina, and pancreas architecture in 400 mg/kg compared to the 200 mg/kg treated group and D25G, thereby restoring fully transparent lenses in diabetes-induced rats. In conclusion, 400 mg/kg PSPL is the most effective dose for the amelioration of STZ-induced DR pathology in male SD rats.

## 1 Introduction

Diabetic retinopathy (DR) is a clinical pathology that arises due to diabetic mellitus and uncontrolled hyperglycemic conditions. The most common clinical signs include visual impairment due to changes in the microvascular structure, lesions in the retina due to the presence of hard or soft exudates, microaneurysms, thickening, and fluid buildup in the retina (Porwal et al., 2018). DR can be classified into proliferative and non-proliferative phases. In the proliferative phase, fragile aberrant vessels start to form, whereas a clear vascular lesion is visibly designated as vascular tortuosity, microaneurysms, or retinal hemorrhages in the non-proliferative phase (Rübsam et al., 2018). According to the prediction by the International Diabetes Federation, there will be approximately 629 million adults with a diabetic condition by the year 2045 (Gadekallu et al., 2020), although the global prevalence rate for DR is about 93 million (Rübsam et al., 2018). Thus, DR has raised public concern throughout the world. Early detection is the best preventative measure for an exacerbation of DR (Gadekallu et al., 2020).

The currently available interventions in treating DR, such as antivascular endothelial growth factor, panretinal laser photocoagulation (PRP), vitrectomy, and corticosteroid injections, are often accompanied by unavoidable side effects. For instance, although antivascular endothelial growth factor can inhibit the growth of abnormal blood vessels, it can lead to blurry vision, photophobia, and floaters (Cox et al., 2021). Meantime, PRP can induce severe pain, peripheral vision loss, decreased contrast sensitivity, choroidal effusions, loss of color, and night vision (Deschler et al., 2014), and vitrectomy can result in iatrogenic retinal breaks and hemorrhages (Brănişteanuonstanti et al., 2016). Hence, natural extracts serve as an alternative intervention for treating and delaying the progression of DR (Taher et al., 2016), (Das et al., 2021). Several plants are under study with the aim of providing a wide reach to cater to cases of DR globally. In China, Chinese herbal medicine alone or combined with laser therapy has been further researched to treat DR in clinical settings (Zhang et al., 2018). For example, purple sweet potato, also known as *Ipomoea batatas* (*I. batatas*), has caught the attention of researchers due to its natural anti-diabetic properties (Escobar-Puentes et al., 2022). It can be consumed raw because all its parts comprise nutrition and bioactive compounds. Essentially, *I. batatas* are significantly rich in lutein compared to other commonly available major greens in Asian countries (Alam, 2021). Lutein is a strong antioxidant and possesses anti-diabetic properties due to its xenobiotic phytochemical constituents. Some of its genotypes are associated with nutraceutical value (Escobar-Puentes et al., 2022). Despite its known functional value, the plant is said to be still under usage due to a lack of scientific data in the therapeutic field. Thus, this study was designed to study the effect of PSPL on the amelioration of DR in streptozotocin (STZ)-induced male Sprague–Dawley (SD) rats.

## 2 Materials and methods

### 2.1 Collection and confirmation of plant species

Fresh purple sweet potato leaves (PSPL) were obtained from a commercial sweet potato farm located at Sungai Pelek, Sepang, Selangor, Malaysia. The leaves were cleaned and sent to the Herbarium Biodiversity Unit, Universiti Putra Malaysia, for confirmation (voucher code: MFI 0188/20).

### 2.2 Ethanol extraction of purple sweet potato leaf

The collected PSPL were cleaned with running tap water to remove any foreign material. Approximately 20 g of PSPL were soaked in 200 ml of 80% ethanol in a conical flask. Then, the mixture was placed in an orbital incubator shaker for 24 h at 150 rpm at room temperature. After that, the supernatant was collected and filtered using Whatman N °1 paper. The same process was repeated three times to obtain maximum yield. All the filtrates from each extraction were combined and evaporated using a rotary evaporator at 48°C. The obtained crude extract was then mixed with maltodextrin in a ratio of 1:1 and oven-dried overnight (Fu et al., 2016). The final extracted powder was weighed and stored at −20 °C until further usage.

### 2.3 Diet-streptozotocin-induced SD diabetic rat model

Male SD rats weighing 150–200 g were randomly divided into two groups. One group (*n* = 10) were fed with a standard rat pellet comprising 306.2 kcal/100 g with 48.8% carbohydrate, 21% protein, and 3% fat, whereas another group (*n* = 40) were fed with a high-fat diet (HFD) pellet comprising 414 kcal/100 g with 43% carbohydrate, 17% of protein, and 40% fat (Abidin et al., 2021). HFD-fed rats were further injected intraperitoneally with a single dose of 60 mg/kg STZ (95% of succession ratio) dissolved in 0.1 mol/L citric acid and 0.1 mol/L sodium citrate with a pH of 4.5. All rats were given free access to food and water, whereas STZ-injected rats were given a 5% glucose solution to drink for the next 24 h to counter fatal hypoglycemia (Ramachandran et al., 2012). Diabetes was confirmed six days after STZ injection using Glucocard™ 01-mini (Arkray Factory, Inc., Japan). Fasting blood glucose (FBG) of ≥11.1 mmol/L was considered in diabetic rats and was selected for the study (Wang et al., 2018).

### 2.4 Experimental groups

The control group and successful diabetes-administered model were treated as follows: normal rats with normal saline (NS), diabetic rats with normal saline (DNS), diabetic rats with oral administration of 25 mg/kg of gliclazide (D25G), diabetic rats with oral administration of 200 mg/kg of PSPL extract (DT 200), and diabetic rats with oral administration of 400 mg/kg of PSPL extract (DT 400). All groups received treatment via oral gavage for 12 weeks. The dose was selected based on the toxicity study by Hisamuddin et al. (2023) on the PSPL extract. Changes in body weight, calorie intake, and 24 h water intake were recorded weekly. At the end of the experiment, all rats were euthanized with CO_2_ overdose; blood was withdrawn using cardiac puncture; and the retina and pancreas were isolated, weighed, and stored for further analysis.

### 2.5 Glycemic parameter: OGTT and FBG

OGTT was performed on all groups of rats on the 12th week of treatment. To perform OGTT, all rats fasted overnight for 12 h. The following day, all rats were challenged with 2 g/kg of glucose solution via oral gavage. Blood was drawn from the rat’s tail, and glucose levels were measured at 0, 30, 60, 120, and 240 min using glucose oxidase–peroxidase reactive strips and a glucometer (Glucocard™ 01-mini, Arkray Factory, Inc., Japan) (Barik et al., 2008). The FBG levels were determined on weeks 5, 10, and 12.

### 2.6 Glycemic parameter: insulin

The DRG Rat Insulin ELISA Kit was used to measure serum and retinal insulin levels. Blood was collected through cardiac puncture and centrifuged for 15 min at 25,000 rpm to obtain serum, which was then used to estimate the serum insulin level (Sayeli and Shenoy, 2021). Meanwhile, the retina was homogenized in lysis buffer using a sonicator to obtain the homogenate, which was then incubated for 30 min. The homogenate was then centrifuged for 10 min at 10,000 rpm, which was then used to estimate the retinal insulin level (Fort et al., 2011).

### 2.7 Interleukin- (IL-) 17A measurement

The level of IL-17A in the retina and serum was assessed using a rat ELISA kit (R&D Systems, Minneapolis, MN, United States) as directed by the manufacturer (Zhu et al., 2022).

### 2.8 Antioxidant parameter

Serum and retina ferric-reducing ability of plasma (FRAP), glutathione (GSH), and total antioxidant capacity (TAC) were measured according to the protocol described for double-antibody sandwich enzyme-linked immunosorbent assay ELISA kits (Cayman Chemical Company, United States) (Agrawal et al., 2012).

### 2.9 Lenticular clarity

Lenticular clarity was performed by anesthetizing rats with 3% isoflurane via inhalation. The visual appearance of cataract formation was viewed under a binocular microscope by administering mydriatic eye drops to all rats. The severity of cataract formation was measured according to the grading system described elsewhere (Chemerovski-Glikman et al., 2018). This was further validated and approved by certified ophthalmologists with the evidence of photographs.

### 2.10 Measurement of retinal thickness

Retinal thickness was measured by dissecting the whole retina in 5% agarose, followed by sectioning at 200 μm using PELCO easiSlicer™. Sectioning containing nerve head extension up to the peripheral edge was selected for thickness analysis. Average thicknesses were determined from the retinal thickness sampled from 100, 200, 300, 400, and 500 μm from the optic nerve head (Toh et al., 2019). The thickness of the retinal blood vessel was measured using fluorescein angiogram negatives and interpreted through a computerized image analyzer according to the protocol described elsewhere (Eaton and Hatchell, 1988). The thickness of the outer nuclear layer was measured at an interval of 480 μm at eight selected regions. Both superior and inferior hemispheres were measured (Huang et al., 2004).

### 2.11 Histopathological analysis

The pancreas was fixed in 10% phosphate buffer formalin and sectioned, and slides were prepared, followed by staining with H&E and observation under a microscope with ×200 magnification. Then, the capture photomicrograph was verified by a certified pathologist from UPM.

### 2.12 Morphometry

Islet specimens were isolated from the histological section and used for morphometric analysis. The islet profiles were then captured using a Leitz DMR microscope. The area of the islet of Langerhans (µm) and the number of β-cells/islets were calculated as *d* = 2 $\sqrt{ab}$ , where *a* and *b* are semidiameters measured at the right and left angles from each islet profile (Morini et al., 2006).

### 2.13 Statistical analysis

Statistical analysis was performed using SPSS version 26.0, and all results were expressed as mean ± standard error of the mean (SEM) for body weight, food consumption, and calorie intake. A normality test was run for all data. One-way ANOVA and the *post hoc* Tukey test were used to analyze the significant differences among groups. A probability of *p* < 0.05 was defined as a statistically significant result.

## 3 Results

### 3.1 Body weight changes and fasting blood glucose after diabetic induction

Table 1 shows the changes in body weight and FBG after diabetic induction. The results indicate a significant reduction in the body weight of DNS after 10 days of successful diabetic induction compared to the NS group. FBG of DNS is significantly higher (*p* < 0.05) than the control group.

**TABLE 1:** Changes in body weight and FBG in SD rats after diabetic induction. Different letters indicate significant differences at *p* < 0.05 among the tested groups. Values are expressed as mean ± SEM.

	Body weight (g)	
	Normal (NS)	Diabetic (DNS)
Day 0	219.50 ± 12.57	225.79 ± 10.72
Day 10	266.17 ± 13.55^a^	216.36 ± 13.99^b^
Fasting blood glucose (mmol/L)		
Day 0	4.13 ± 0.17	3.79 ± 0.20
Day 10	3.67 ± 0.19^a^	15.52 ± 0.66^b^

### 3.2 Body weight changes and fasting blood glucose after treatment

Table 2 presents the changes in body weight and FBG after treatment with different concentrations of the PSPL extract. The body weights of the DNS, DT 200, DT 400, and D25G groups are significantly lower (*p* < 0.05) than those of the NS group on week 12. However, on week 12, there is no significant difference (*p* > 0.05) between the DNS, DT 200, DT 400, and D25G groups in body weight. For the FBG level, there is no significant difference (*p* > 0.05) between the NS, DNS, DT 200, DT 400, and D25G groups on week 5. There is a significant increase (*p* < 0.05) in the FBG level in the DNS, DT 200, DT 400, and D25G groups compared to the NS group on weeks 10 and 12. However, on week 12, there is a significant decrease (*p* < 0.05) in the FBG level of the DT 400 group compared to the DNS group.

**TABLE 2:** Effect of different concentrations of PSPL extracts on body weight and FBG of diabetic rats during the 12-week treatment phase. Different letters indicate significant differences at *p* < 0.05 among the tested groups. Values are expressed as mean ± SEM.

	Body weight (g)				
	NS	DNS	DT 200	DT 400	D25G
Week 12	490.17 ± 29.36^a^	307.33 ± 54.24^b^	319.80 ± 57.41^b^	281.00 ± 75.93^b^	211.80 ± 46.53^b^
Fasting blood glucose (mmol/L)					
Week 5	4.63 ± 0.57	15.82 ± 2.59 ^b^	15.34 ± 0.65 ^b^	13.94 ± 3.87 ^b^	16.26 ± 2.96 ^b^
Week 10	3.87 ± 0.08^a^	15.73 ± 1.86^b^	17.16 ± 1.66^b^	19.47 ± 0.80^b^	16.12 ± 2.81^b^
Week 12	4.83 ± 0.23^a^	19.25 ± 2.13^b^	17.82 ± 2.89^bc^	13.27 ± 0.92^c^	14.34 ± 0.78^bc^

### 3.3 Total calorie and water intake in PSPL-treated diabetic rats

Table 3 shows the calorie intake and 24 h water intake in diabetic rats on week 12. The data show a significantly high (*p* < 0.05) amount of water intake in the DNS, DT 200, DT 400, and D25G groups compared to the NS group. For total calorie intake, the DNS group shows significantly high (*p* < 0.05) calorie intake compared to the NS group. In contrast, the DT 200, DT 400, and D25G groups show significantly low (*p* < 0.05) calorie intake compared to the DNS group. However, there is no significant difference (*p* > 0.05) in calorie intake between the DT 200, DT 400, D25G, and NS groups.

**TABLE 3:** Effect of different concentrations of PSPL extracts on fluid and calorie intake of diabetic rats during the 12-week treatment phase. Different letters indicate significant differences at *p* < 0.05 among the tested groups. Values are expressed as mean ± SEM.

	Fluid intake (ml)				
	NS	DNS	DT 200	DT 400	D25G
Week 12	3329.67 ± 172.20^a^	10658.08 ± 956.27^b^	8764.78 ± 623.93^b^	8263.89 ± 342.95^b^	7630.00 ± 629.66^b^
Calorie intake (kJ)					
Week 12	3329.67 ± 172.20^a^	10658.08 ± 956.27^b^	8764.78 ± 623.93^b^	8263.89 ± 342.95^b^	7630.00 ± 629.66^b^

### 3.4 Oral glucose tolerance test in PSPL-treated diabetic rats

**FIGURE 1:**
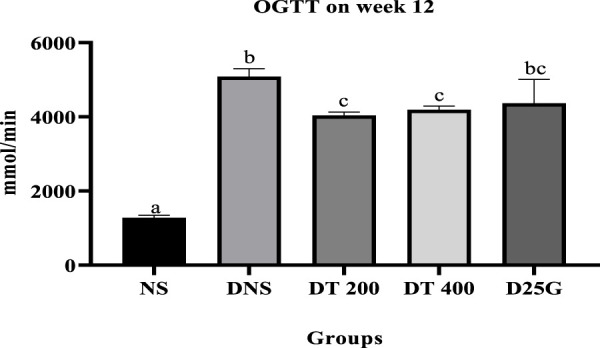
Effect of different concentrations of PSPL extract on the plasma glucose level represented as the area under the curve (AUC) during the 12-week treatment phase. Different letters indicate significant differences at *p* < 0.05 among the tested groups.

Figure 1 shows the OGTT of different concentrations of the PSPL extract during the 12-week treatment phase. Data indicate that the OGTT results of the DNS, DT 200, DT 400, and D25G groups are significantly higher (*p* < 0.05) than those of the NS group. Meanwhile, the OGTT results of the DT 200 and DT 400 groups are significantly lower (*p* < 0.05) than those of DNS. However, the OGTT results of the D25G and DNS groups are similar.

### 3.5 Relative organ weight (ROW) of PSPL-treated diabetic rats

Table 4 presents the ROW and standardized organ weight (%) for diabetic-induced rats. Data show no significant difference (*p* > 0.05) in the ROW of the retina and kidney between the NS, DNS, DT 200, DT 400, and D25G groups. The ROW of the DT 200 and D25G groups for the liver is significantly lower (*p* < 0.05) than that of the DNS group, whereas the ROW of the pancreas of the DNS group is significantly lower (*p* < 0.05) than that of the NS group. However, there is no significant difference (*p* > 0.05) in the ROW of the liver for the DNS, DT 200, DT 400, and D25G groups compared to the NS group. Furthermore, for ROW of the pancreas, there is no significant difference (*p* < 0.05) between the NS, DT 200, DT 400, and D25G groups. The standardized organ weight (%) of the retina showed no significant difference between the NS, DNS, DT 200, DT 400, and D25G groups. The standardized organ weight (%) of the liver for the DNS, DT 200, DT 400, and D25G groups is significantly higher (*p* < 0.05) than that of the NS group. The organ weight (%) of the kidney of the DT 400 group is significantly higher (*p* < 0.05) than that of the NS group. However, there is no significant difference (*p* > 0.05) in the kidneys between the NS, DNS, DT 200, and D25G groups. The standardized organ weight (%) of the pancreas of the DT 200 group is significantly higher (*p* < 0.05) than that of the DNS group. However, there is no significant difference (*p* > 0.05) in the pancreas of the DNS, DT 200, DT 400, and D25G groups compared to the NS group.

**TABLE 4:** Effect of different concentrations of PSPL extracts on ROW and the percentage of organ weight of diabetic rats during the 12-week treatment phase. Different letters indicate significant differences at *p* < 0.05 among the tested groups. Values are expressed as mean ± SEM.

	NS	DNS	DT 200	DT 400	D25G
Relative organ weight (g)					
Retina	0.38 ± 0.04	0.29 ± 0.01	0.28 ± 0.01	0.30 ± 0.03	0.33 ± 0.03
Liver	13.82 ± 0.93^ab^	18.17 ± 1.08^b^	12.88 ± 1.45^a^	13.19 ± 1.60^ab^	11.66 ± 0.61^a^
Kidney	3.33 ± 0.16	3.68 ± 0.23	3.19 ± 0.23	3.07 ± 0.49	2.44 ± 0.17
Pancreas	1.26 ± 0.13^a^	0.59 ± 0.04^b^	0.82 ± 0.210^ab^	0.77 ± 0.13^ab^	0.66 ± 0.20^ab^
Percentage per body weight (%)					
Retina	0.08 ± 0.01	0.07 ± 0.01	0.11 ± 0.02	0.11 ± 0.03	0.14 ± 0.01
Liver	3.08 ± 0.13^a^	4.46 ± 0.09^b^	4.64 ± 0.27^b^	4.37 ± 0.13^b^	4.60 ± 0.51^b^
Kidney	0.74 ± 0.03^a^	0.90 ± 0.04^a^	0.09 ± 0.05^a^	1.00 ± 0.06^b^	0.95 ± 0.10^a^
Pancreas	0.28 ± 0.02^ab^	0.15 ± 0.02^b^	0.29 ± 0.06^a^	0.26 ± 0.03^ab^	0.21 ± 0.03^ab^

### 3.6 Blood biochemistry analysis in PSPL-treated diabetic rats

Table 5 presents data for blood biochemistry analysis of diabetes-induced rats. There is no significant difference (*p* > 0.05) in potassium, urea, and creatinine levels between the NS, DNS, DT 200, DT 400, and D25G groups. The sodium level is significantly lower (*p* < 0.05) in the DNS, DT 200, and D25G groups compared to the NS and DT 400 groups. However, there is no significant difference (*p* > 0.05) in the sodium level between the NS and DT 400 groups. The chloride level is significantly lower (*p* < 0.05) in the DT 400 and D25G groups compared to the NS, DNS, and DT 200 groups. However, there is no significant difference (*p* > 0.05) in chloride levels between the NS, DNS, and DT 200 groups. The liver function test shows no significant difference (*p* > 0.05) in the total protein, albumin, globulin, albumin–globulin ratios, AST, ALT, and GGT levels between the NS, DNS, DT 200, DT 400, and D25G groups. The ALP level is significantly increased (*p* < 0.05) in the DNS and D25G groups compared to the NS, DT 200, and DT 400 groups. However, there is no significant difference (*p* > 0.05) in the ALP level between the NS, DT 200, and DT 400 groups.

**TABLE 5:** Effect of different concentrations of PSPL extracts on blood biochemistry analysis of diabetic rats during the 12-week treatment phase. The different letter indicates a significant difference at *p* < 0.05 among the tested groups. Values are expressed as mean ± SEM.

	NS	DNS	DT 200	DT 400	D25G
Renal function test					
Sodium (mmol/L)	147.50 ± 0.56^a^	141.17 ± 1.08^b^	140.60 ± 1.17^b^	142.67 ± 0.76^a^	140.00 ± 3.42^b^
Potassium (mmol/L)	7.77 ± 0.45	9.23 ± 0.46	8.80 ± 0.37	7.98 ± 0.15	8.46 ± 0.48
Chloride (mmol/L)	100.83 ± 0.70^a^	92.83 ± 1.58^a^	95.80 ± 3.07^a^	92.33 ± 0.61^b^	91.25 ± 4.03^b^
Urea (mmol/L)	6.57 ± 0.52	5.58 ± 0.90	8.43 ± 1.32	8.20 ± 0.32	6.04 ± 1.26
Creatinine (mmol/L)	50.00 ± 3.60	58.67 ± 3.91	52.75 ± 2.95	59.50 ± 2.27	58.20 ± 3.01
Liver function test					
Total protein (g/L)	66.83 ± 2.09	68.33 ± 2.74	63.80 ± 1.83	68.17 ± 2.01	64.60 ± 3.50
Albumin (g/L)	38.67 ± 1.37	39.33 ± 1.38	35.40 ± 0.68	37.00 ± 0.69	36.00 ± 1.48
Globulin (g/L)	28.17 ± 0.86	29.00 ± 1.65	28.40 ± 1.36	31.17 ± 1.53	28.60 ± 2.64
Albumin–globulin ratio	1.37 ± 0.03	1.36 ± 0.06	1.26 ± 0.06	1.21 ± 0.04	1.29 ± 0.10
ALP (U/L)	108.17 ± 13.84^a^	701.75 ± 147.17^b^	336.00 ± 69.06^a^	163.00 ± 17.09^a^	899.75 ± 223.26^b^
AST (U/L)	120.80 ± 5.63	89.33 ± 10.70	132.20 ± 21.09	131.83 ± 21.10	249.20 ± 92.64
ALT (U/L)	77.00 ± 8.50	139.00 ± 21.65	128.00 ± 22.21	114.50 ± 24.08	313.80 ± 159.39
Gamma-glutamyl transferase (U/L)	2.67 ± 0.56	2.67 ± 0.33	4.25 ± 0.22	4.00 ± 1.15	3.60 ± 0.40

### 3.7 Serum and retinal insulin levels in PSPL-treated diabetic rats

**FIGURE 2:**
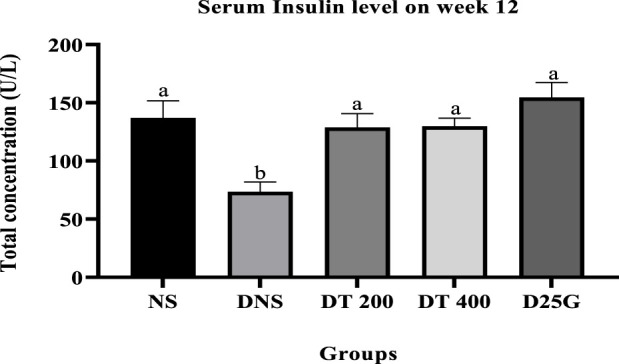
Effect of different concentrations of PSPL extracts on the serum insulin level during the 12-week treatment phase. Different letters indicate significant differences at *p* < 0.05 among the tested groups.

**FIGURE 3:**
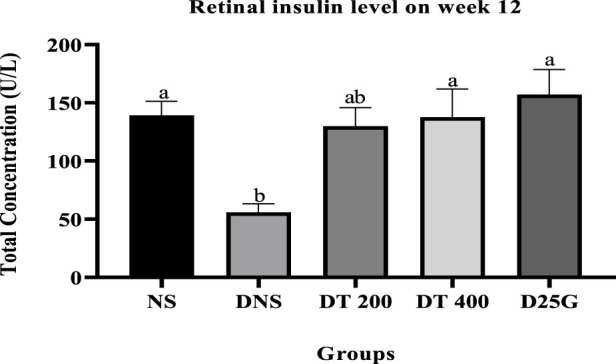
Effect of different concentrations of PSPL extracts on the retinal insulin level during the 12-week treatment phase. Different letters indicate significant differences at *p* < 0.05 among the tested groups.

Figures 2, 3 show the serum and retinal insulin levels in diabetic rats of different concentrations of the PSPL extract during the 12-week treatment phase. The serum insulin of the DNS group is significantly lower (*p* < 0.05) than that of the NS, DT 200, DT 400, and D25G groups. However, there is no significant difference (*p* > 0.05) in the serum insulin level between the NS, DT 200, DT 400, and D25G groups. The retinal insulin level of the DNS group is significantly lower (*p* < 0.05) than that of the NS, DT 400, and D25G groups. However, there is no significant difference (*p* > 0.05) in the retinal insulin level between the NS, DT 400, and D25G groups.

### 3.8 IL-17A in the retina and serum

**FIGURE 4:**
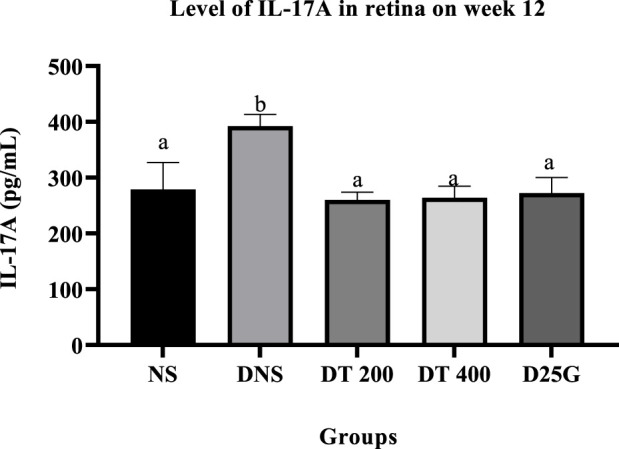
Effect of different treatments of PSPL extracts on the IL-17A level in the retina of diabetes-induced rats after the 12-week treatment phase. The different letter indicates a significant difference at *p* < 0.05 among the tested groups.

**FIGURE 5:**
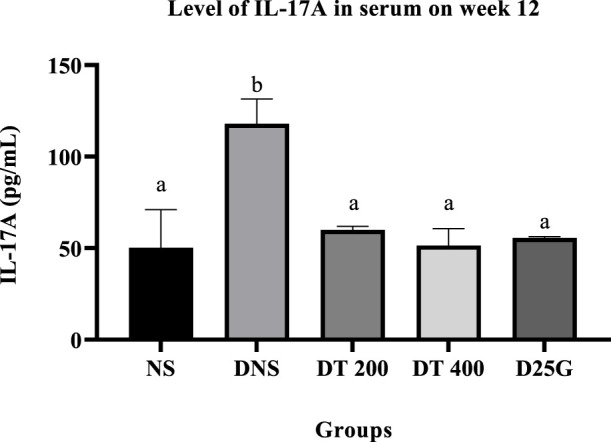
Effect of different treatments of PSPL extracts on the IL-17A level in the serum of diabetes-induced rats after the 12-week treatment phase. The different letter indicates a significant difference at *p* < 0.05 among the tested groups.

Figures 4, 5 present the effect of different treatments of PSPL extract on IL-17A on serum and retina in diabetic-induced rats. Data show that the level of IL-17A is significantly increased (*p* < 0.05) in the serum and retina of the DNS group compared to the NS, DT 200, DT 400, and D25G groups. However, there is no significant difference (*p* > 0.05) in the IL-17A level in serum and retina between the NS, DT 200, DT 400, and D25G groups.

### 3.9 Antioxidant profile of PSPL-treated diabetic rats

**FIGURE 6:**
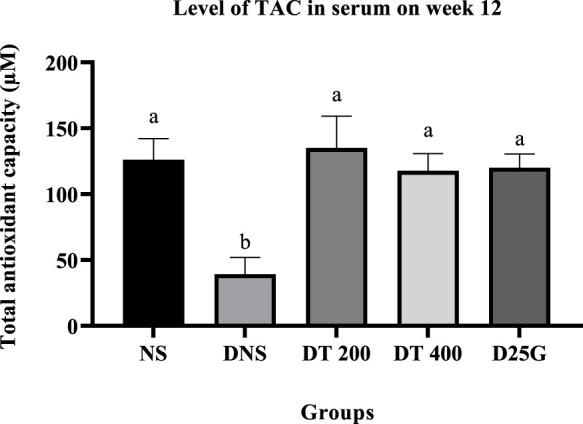
Effect of different treatments of PSPL extract on the TAC level in the serum of diabetes-induced rats after the 12-week treatment phase. The different letter indicates a significant difference at *p* < 0.05 among the tested groups.

**FIGURE 7:**
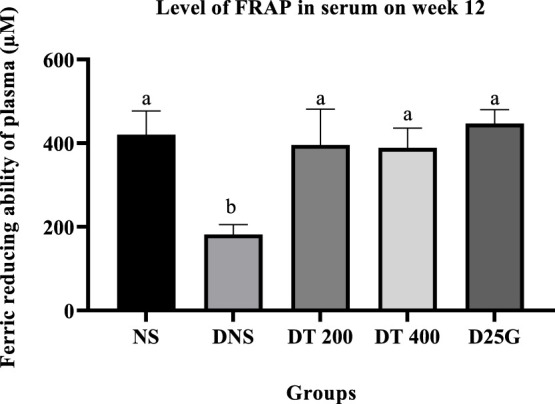
Effect of different treatments of PSPL extract on the FRAP level in the serum of diabetes-induced rats after the 12-week treatment phase. The different letter indicates a significant difference at *p* < 0.05 among the tested groups.

**FIGURE 8:**
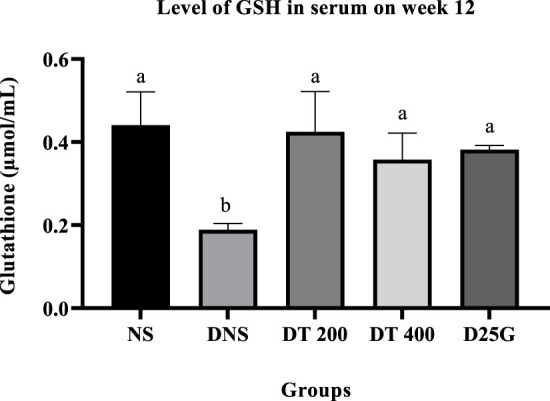
Effect of different treatments of PSPL extract on the GSH level in the serum of diabetes-induced rats after the 12-week treatment phase. The different letter indicates a significant difference at *p* < 0.05 among the tested groups.

**FIGURE 9:**
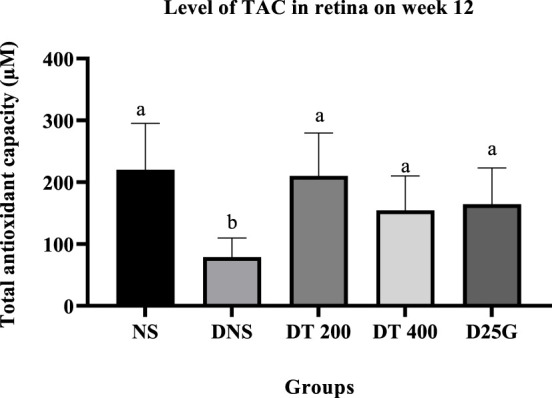
Effect of different treatments of PSPL extract on the TAC level in the retina of diabetes-induced rats after the 12-week treatment phase. The different letter indicates a significant difference at *p* < 0.05 among the tested groups.

**FIGURE 10:**
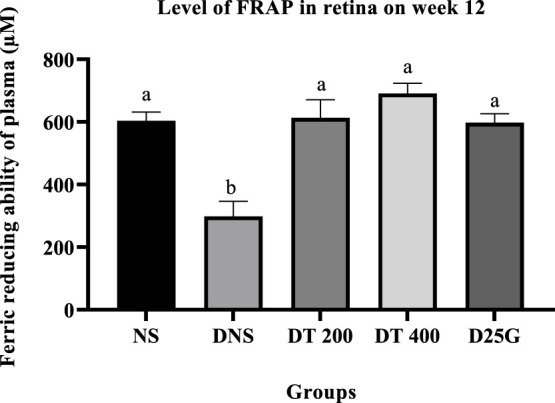
Effect of different treatments of PSPL extract on the FRAP level in the retina of diabetes-induced rats after the 12-week treatment phase. The different letter indicates a significant difference at *p* < 0.05 among the tested groups.

**FIGURE 11:**
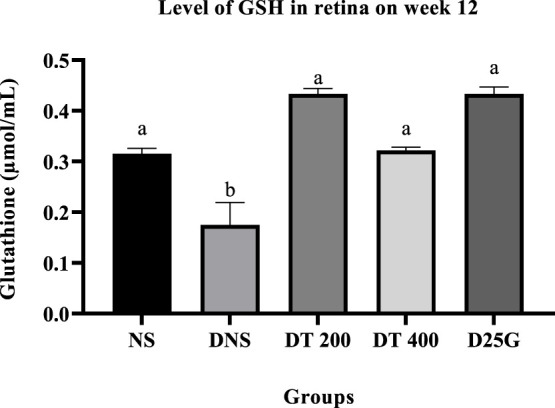
Effect of different treatments of PSPL extract on the GSH level in the retina of diabetes-induced rats after the 12-week treatment phase. The different letter indicates a significant difference at *p* < 0.05 among the tested groups.

Figures 6–11 present the effect of different treatments of PSPL extract on TAC, FRAP, and GSH levels on serum and retina in diabetes-induced rats. Data show that the levels of TAC, GSH, and FRAP are significantly high (*p* < 0.05) in the serum and retina of the NS, DT 200, DT 400, and D25G groups compared to the DNS group. However, there is no significant difference (*p* > 0.05) in TAC, GSH, and FRAP levels in serum and retina between the NS, DT 200, DT 400, and D25G groups.

### 3.10 Retinal thickness of PSPL-treated diabetic rats

**FIGURE 12:**
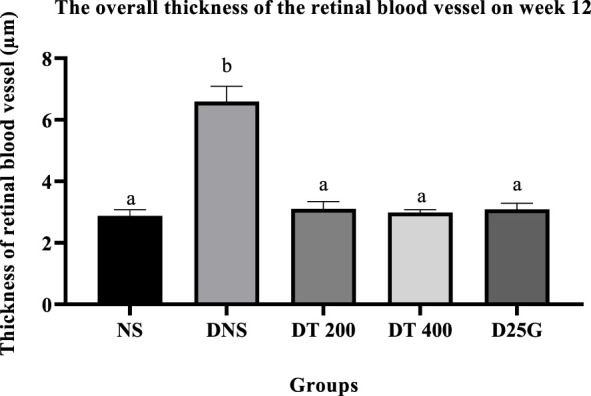
Effect of different treatments of PSPL extract on the thickness of blood vessels of diabetes-induced rats after the 12-week treatment phase. The different letter indicates a significant difference at *p* < 0.05 among the tested groups.

**FIGURE 13:**
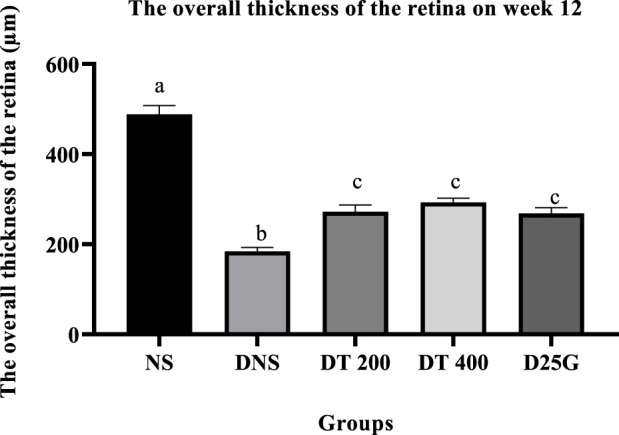
Effect of different treatments of PSPL extract on the overall thickness of the retina of diabetes-induced rats after the 12-week treatment phase. The different letter indicates a significant difference at *p* < 0.05 among the tested groups.

**FIGURE 14:**
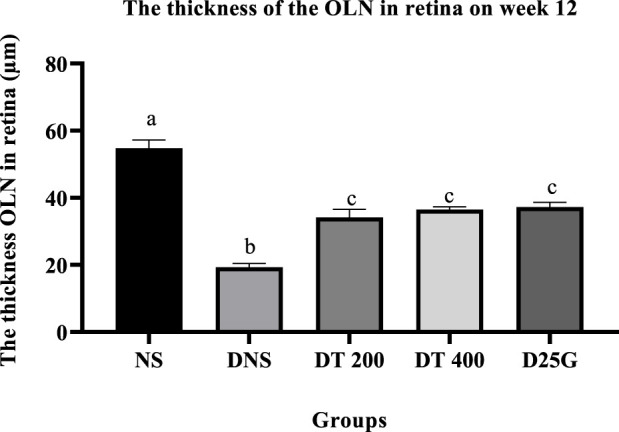
Effect of different treatments of PSPL extract on the outer nuclear layer (OLN) of diabetes-induced rats after the 12-week treatment phase. The different letter indicates a significant difference at *p* < 0.05 among the tested groups.

Figures 12–14 show the overall thickness of the retina blood vessel, the thickness of the outer layer of the retina nucleus (OLN), and the overall thickness of the retina. Data indicate that the retinal blood vessel of the DNS group is significantly higher (*p* < 0.05) compared to the NS, DT 200, DT 400, and D25G groups. However, there is no significant difference (*p* > 0.05) in retinal blood vessel thickness between the NS, DT 200, DT 400, and D25G groups. Meanwhile, overall retina and OLN thicknesses are significantly lower (*p* < 0.05) in the DNS, DT 200, DT 400, and D25G groups compared to the NS group. However, overall retina thickness and OLN thickness are significantly greater (*p* < 0.05) in the DT 200, DT 400, and D25G groups compared to the DNS group.

### 3.11 Lenticular clarity of PSPL-treated diabetic rats

Supplementary Figure S1 represents the effect of different treatments of PSPL extract on cataract formation in diabetic rats after the 12-week treatment phase. The control group (NS) shows a fully transparent lens, whereas the DNS group shows a severe appearance of lenticular opacification. Meanwhile, in the DT 200, DT 400, and D25G groups, a delayed process of lenticular opacification with a reduced severity level of cataract is seen. PSPL extract enhances lens clarity and slows down diabetic-induced cataract formation.

### 3.12 Histopathological analysis of PSPL-treated diabetic rats

Supplementary Figure S2 presents the histological section of the retina and pancreas of diabetic-induced rats. The histological section of the retina for the DNS group shows the vitreoretinal interface in the retina and loss of neurons in the GCL, presence of cystoid spaces in the INL and OPL, hyperreflective foci in the ONL of the retina, and degeneration and atrophy in the PL and pool of extracellular fluid at the RPE of the retina. However, the DT 200, DT 400, and D25G groups show the normal histological section of GCL, IPL, INL, OPL, ONL, PL, and RPE, identical to the NS group. The histology of the pancreas shows degeneration and atrophy in the central region of the islet of Langerhans in the DNS group. Normal histological structure of islets of Langerhans and acini is observed in the pancreas of the NS group. Similarly, the histology of the pancreas of the DT, DT 400, and D25G groups shows the normalization of the islet of the Langerhans area that is identical to the NS histology.

### 3.13 Morphometry

Table 6 shows the morphometry analysis of the area of the islet of Langerhans and the number of β-cells/islets in diabetic rats after the 12-week treatment phase. Data show that the area of the islet of Langerhans and the number of β-cells/islets in the DNS group are significantly lower (*p* < 0.05) compared to the NS, DT 200, DT 400, and D25G groups. However, there is no significant difference (*p* > 0.05) in the area of the islet of Langerhans and the number of β-cells/islets between the NS, DT 200, DT 400, and D25G groups.

**TABLE 6:** Effect of different treatments of PSPL extract on the area of the islet of Langerhans and the number of β-cells/islets of diabetes-induced rats after the 12-week treatment phase. The different letter indicates a significant difference at *p* < 0.05 among the tested groups. Values are expressed as mean ± SEM.

	Islet of Langerhans				
	NS	DNS	DT 200	DT 400	D25G
Area of the islet of Langerhans (µm)	2.17 ± 0.23^a^	0.65 ± 0.07^b^	1.66 ± 0.31^a^	1.90 ± 0.31^a^	1.78 ± 0.16^a^
Number of β-cells/islets	187.83 ± 23.73^a^	59.83 ± 6.89^b^	163.83 ± 22.87^a^	135.83 ± 20.57^a^	144.17 ± 19.18^a^

## 4 Discussion

PSPL is rich in lutein, a strong antioxidant (Cavalier et al., 2019), possesses a beneficial effect as an anti-inflammatory agent, and can reverse cataract formation (Buscemi et al., 2018). Thus, it has been speculated that the lutein compound can prevent and delay the progression of DR (Sahli et al., 2015). Accordingly, this study was designed to study the effect of PSPL on the amelioration of DR pathology in STZ-induced male SD rats. Retroperitoneal injection of STZ successfully resulted in diabetic induction by day 10, as shown in Table 1. Drastic weight loss in the DNS group (Skovsø, 2014) and FBG concentration of >11.1 (Wu et al., 2020) with a single dose of STZ administration proved the successful establishment of the diabetic model. Following confirmation of diabetes, the rats were administrated with different concentrations of the PSPL extract (DT 200 and DT 400) for 12 weeks. During the 12-week follow-up, the body weight of the DNS group remained low compared to the NS group, proving the theory that excessive weight loss is common clinical pathology in diabetic subjects (Galicia-Garcia et al., 2020). However, a slight increase in body weight in the DT 200 group showed that the PSPL extract might have little effect on reversing drastic weight loss in diabetic rats. However, the body weights of the DT 400 and D25G groups remained lower than the DNS group, indicating that the PSPL extract did not affect body weight. Comparably, the PSPL extract did not affect total water and calorie intake in treated and untreated groups compared to the NS group. Similarly, the FBG of all treated and untreated groups up to weeks 5 and 10 remained extravagantly high compared to the NS group. However, in week 15, the significant reduction in the DT 200 and DT 400 groups showed the ability of lutein-rich PSPL extract as an anti-hyperglycemic agent. Data are further supported by Sharavana et al. (2017).

The ROW and percentage per body weight of the liver in the DNS group are heavier due to the presence of high-level fat accumulation in the liver, which supports the theory of successful diabetic induction in rats (Bozzetto et al., 2010), causing the ROW of the liver to rise. Over time, chronic exposure to fats in the liver will cause the spillage of fats into the pancreas. Excessive deposition of fats into the pancreas will hinder the normal function of β-cells (Li et al., 2020), (Yahya et al., 2013). This is the underlying reason for the significantly reduced levels of serum and retinal insulin in the DNS group. Similarly, excessive calorie intake in diabetic subjects will enhance fat delivery to the islet cells in the pancreas, resulting in intracellular accumulation of triglycerides in the long term. Persistent addition of triacylglycerol will stimulate the intrapancreatic to be replaced by adipocytes (Yu and Wang, 2017), altering the normal insulin secretion of β-cells. In contrast, a reduced level of insulin production by β-cells will cause the volume of the pancreas to shrink (Al-Mrabeh et al., 2016). Thus, the ROW of the pancreas of the DNS group appears significantly low compared to the NS group. Kidney enlargement is another common complication of diabetes (Sharavana et al., 2017). Nevertheless, a modest decrease in kidney size in the DT 200 and DT 400 groups indicates the protective effects of lutein in maintaining kidney architecture (Liu et al., 2014). However, the significant reduction of ROW of the liver in the DT 200, DT 400, and D25G groups could result from the effect of high lutein concentration in PSPL extract because lutein can promote fat loss (Hajizadeh-Sharafabad et al., 2020), thereby reversing the action of insulin insensitivity in the pancreas. As a result, the ROW of the pancreas in the DT 200, DT 400, and D25G groups is similar to that of the NS group.

Paradoxically, a rise in serum osmolality (osmotic diuresis) that eventually results in a reduced sodium level by dilution (Liamis et al., 2014) is a common feature in diabetes, which may influence the concentration of the serum chloride level (Kataoka and Yoshida, 2020). A similar value of DT 400 of the sodium level with NS and a decrease in chloride content in serum prove that lutein can balance serum electrolytes (Sindhu and Kuttan, 2013) in diabetes-induced rats. An abnormal increase in ALP is a direct reflection of the increase in ROW in kidney and kidney dysfunction in diabetics as amino acids are converted into ketoacids in hyperglycemic conditions (Zakaria et al., 2019; Konda et al., 2019). In such a case, lutein content in PSPL suppresses the excessive stimulation of protein catabolism (Mrowicka et al., 2022) and represses gluconeogenesis through the Sirtuin 1 pathway (Hwang et al., 2018; Zang et al., 2018). However, gliclazide is less effective for treating diabetes complications. Thus, the ALP level remains high despite treatment in the D25G group (LiverTox, 2018).

Additionally, the hyperglycemic condition in DNS activates a metabolic pathway involving diacylglycerol, protein kinase C, and nicotinamide adenine dinucleotide phosphate (NADPH) (Rasheed et al., 2022). Accordingly, the formation of reactive oxygen species (ROS) and oxidative stress rises (Müller et al., 2011). Thus, the levels of FRAP, GSH, and TAC are significantly higher in the retina and serum of the DNS group than those in the NS group, leading to microvascular complications, such as the formation of cataracts in diabetes. This is mainly because the imbalance in oxidative stress solubilizes the proteins in the lens, increasing the lens opacity (Kaur et al., 2012). However, the presence of conjugated double bonds and hydroxyl groups in lutein stimulates the antioxidant defense mechanism here by hindering the degree of oxygen penetrating the membranes (Sindhu et al., 2010), thereby reducing and restoring the levels of FRAP, GSH, and TAC in PSPL-treated groups (DT 200 and DT 400). In addition, diabetes increases the expression of IL-17A in the retina and serum. IL-17A is a proinflammatory cytokine of T helper type 17 cells, leading to vascular damage in the retina by inducing retinal apoptosis (Qiu et al., 2016). IL-17A promotes retinal inflammation and damage by inducing the production of proinflammatory cytokines, such as IL-1β, IL-6, and TNF-α (Byrne et al., 2021), and by increasing oxidative stress and vascular permeability in the retina (Sigurdardottir et al., 2019). IL-17A also contributes to the breakdown of the blood–retinal barrier (BRB), which is a protective barrier that helps maintain the proper environment for the retina to function (Zhong and Sun, 2022). In DR, the breakdown of the BRB can lead to the leakage of fluids and blood into the retina, which can contribute to retinal damage and vision loss (Rudraraju et al., 2020). Thus, this hypothesis is proven in this study, where the laboratory analysis shows that IL-17A is significantly elevated in the DNS group compared to the NS group. However, the lutein antioxidant acts as a micronutrient in such conditions by reducing the inflammatory response (Sindhu et al., 2010), as shown in PSPL-treated groups (DT 200 and DT 400).

In addition, the change in the diameter of the retinal blood vessel, its overall thickness, and OLN is a key predictor for the progression of DR, a main complication of diabetes mellitus (Bek, 2017). This is mainly because the accumulation of lactate in the hyperglycemic condition and retinal hypoxia stimulates the blood vessel of the retina, which increases the diameter (Klein et al., 2011) and makes it prone to microvascular damage, probably reducing the overall thickness of the retina over time and causing severe central vision loss (Alghadyan, 2011), as shown in the DNS group. Thus, this altered neuroretinal degeneration further enhances the thinning of OLN (Ishibashi and Tavakoli, 2020) in the DNS group. In the bargain, in diabetes, an excess glucose level in aqueous humor stimulates aldose reductase, a catalyzing enzyme that converts glucose to sorbitol. However, excessive sorbitol levels cannot diffuse out of the lens or cell membranes, resulting in the accumulation of sorbitol in the lens, thereby generating an osmotic gradient (Srinivasan and Preedy, 2014). As a result of this unregulated osmotic stress, ROS is formed (Martinière et al., 2019), and apoptosis is induced in the epithelial cell’s lens (Pollreisz and Schmidt-Erfurth, 2010), causing water to diffuse into the lens, leading to cataract formation (Srinivasan and Preedy, 2014). However, treatments with the lutein-rich PSPL extract (DT 200 and DT 400) and a controlled drug (gliclazide) in this study decrease the thickness of blood vessels and increase the thickness of the overall retina and ONL. Furthermore, lutein-rich PSPL neutralizes ROS and reverses ROS-induced cataract formation in the lens (Maci and Santos, 2015), as shown in the DT 200 and DT 400 groups.

Therewithal, drastic loss of β-cells directly influences the decreased level of the area of islets in Langerhans in diabetic subjects (Yagihashi et al., 2016), as shown in the DNS group. The hyperglycemic condition hinders the ability of β-cells to differentiate (Weir et al., 2013), causing trans-differentiation or de‐differentiation of β‐cells into α‐cells, thereby reducing the volume of islets (Yagihashi et al., 2016). Anyhow, treatments with the lutein-rich PSPL extract (DT 200 and DT 400) and a controlled drug (gliclazide) reverse the loss of β-cells and restore the volume of islets in Langerhans in diabetic rats, as shown in the histological section of the pancreas. The presence of vitreoretinal interface in the retina and loss of neurons in the GCL, cystoid spaces in the INL and OPL, hyperreflective foci in the ONL of the retina, degeneration and atrophy in the PL, and pool of extracellular fluid at the RPE of the retina is another pathological change observed in the retinal histology section of the DNS group, as shown in Figure 9. Treatment with the lutein-rich PSPL extract (DT 200 and DT 400) prevents histological changes in the retina of diabetic-induced rats to a significant extent, and the retina shows similar histological features to the NS group, proving that the PSPL extract can ameliorate DR pathology.

## 5 Conclusion

In the present study, the lutein-rich PSPL extract ameliorated DR pathology. The lutein-rich PSPL supplementation restored the FBG, sodium, chloride, ALP, and insulin levels within the normal range in diabetes-induced rats. In addition, lutein exhibited protective effects in maintaining the kidney, liver, retina, and pancreas architecture in the 400 mg/kg treated group. Being a strong antioxidant, lutein restored the levels of FRAP, GSH, and TAC and reduced the level of IL-17A in the serum and retina of diabetic rats. The overall thicknesses of the retina, blood vessel, and ONL were reduced to a similar value to non-diabetic rats in the 400 mg/kg treated group, thereby restoring fully transparent lenses in diabetes-induced rats. In conclusion, the current data show that lutein-rich PSPL supplementation of the 400 mg/kg dosage is an effective dose for the amelioration of DR in STZ-induced SD rats.

## 6 Limitations and future directions

Based on the findings of our study, several limitations and potential future directions can be explored. One potential direction is to investigate the long-term effects of lutein-rich PSPL supplementation on DR pathology in diabetic rats. This could involve extending the treatment period beyond the 12 weeks used in the current study to determine whether the protective effects of PSPL are sustained over a longer period of time. Another direction could be to investigate the signaling pathway underlying the protective effects of PSPL on DR pathology, focusing on the proteomic expression on the retina. This might give insight into a more targeted therapeutic focus in DR. Additionally, future studies could investigate the safety and efficacy of PSPL extract on humans with DR. This could involve conducting clinical trials to determine optimal dosages, assess any potential side effects, and evaluate the effectiveness of PSPL extract in reducing DR pathology in human patients. Overall, the findings of this study suggest that PSPL extract can be a therapeutic agent for DR, and further research is warranted to fully explore its potential benefits and mechanisms of action.

## Data Availability

The raw data supporting the conclusion of this article will be made available by the authors, without undue reservation.
