# Supplementary material for: The role of lutein-rich purple sweet potato leaf extract on the amelioration of diabetic retinopathy in streptozotocin-induced Sprague–Dawley rats
Source: Front Pharmacol. 2023 May 18;14:1175907. doi: 10.3389/fphar.2023.1175907 (PMC10232805; doi:10.3389/fphar.2023.1175907)
Supplement: Supplementary file 1 [file Presentation1.pdf]

Supplementary Material

The role of lutein-rich purple sweet potato leaf extract on the amelioration of diabetic retinopathy in streptozotocin-induced Sprague Dawley rats

Ahmad Safiyyu'd-din Bin Hisamuddin 1,2, Ruth Naomi 3, Khairul Aiman Bin Manan 2, Hasnah Bahari 3, Fezah Othman 2, Hashim Embong 4, Amin Ismail 5, Qamar Uddin Ahmed 6, Siti Hadizah Jumidil 3, Mohd Khairi Hussain 2*, Zainul Amiruddin Zakaria 1*

Correspondence: *

1 Mohd Khairi Hussain, khairi@upm.edu.my (M.K.H)

2 Zainul Amiruddin Zakaria, zaz@ums.edu.my (Z.A.Z)

Organ	NS	DNS	DT 200	DT 400	D25G
Retina	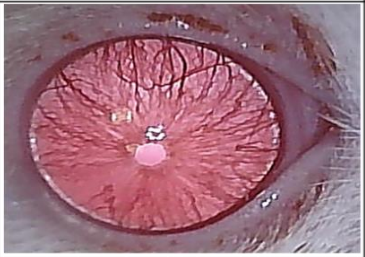	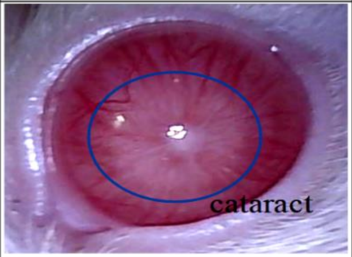	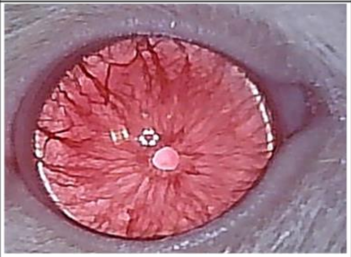	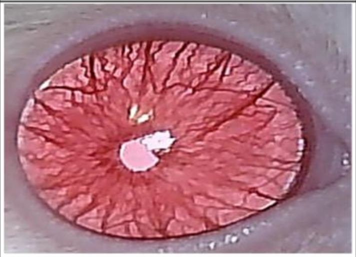	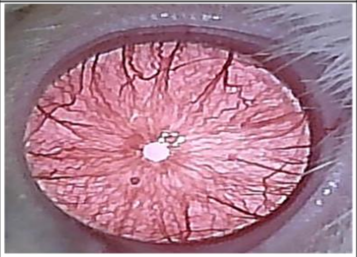

Figure S1. Photograph of diabetic-induced rat's retina. NS and D25G display fully transparent lenses while DT 200, and DT 400 photographs of diabetic rats with clear lenses treated with PSPL extract.

Organ	NS	DNS	DT 200	DT 400	D25G
Retina	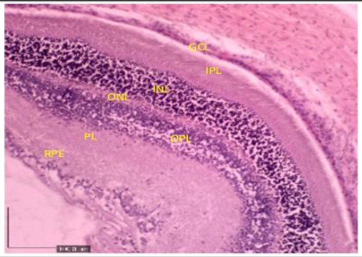	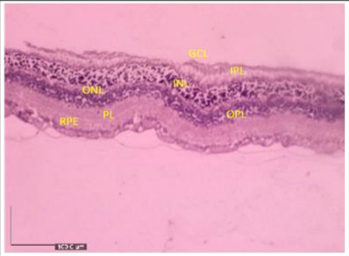	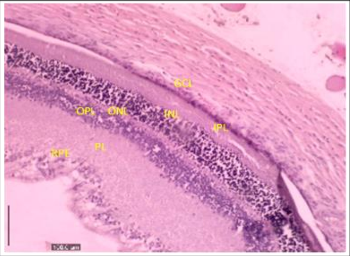	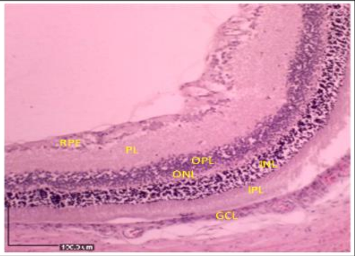	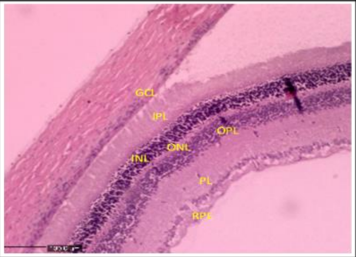
Pancreas	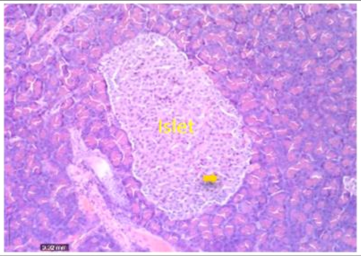	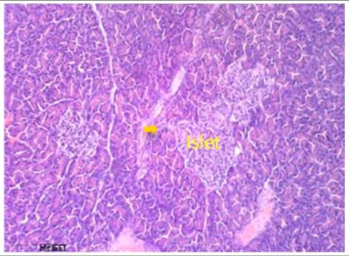	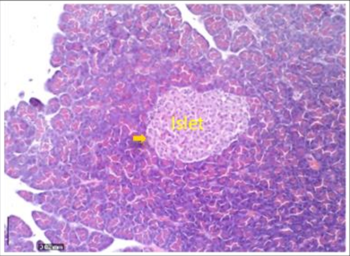	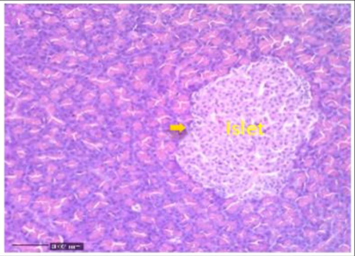	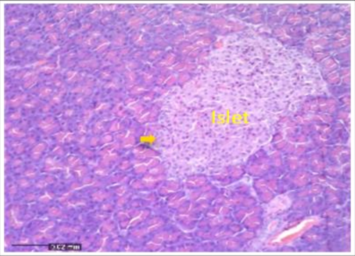

Figure S2. Histology section of diabetic rats treated with PSPL extract after the 12-week treatment phase. DT 200, DT 400, and D25G shows histological section identical to the NS group. DNS group shows the vitreoretinal interface in the retina and loss of neurons in the GCL, presence of cystoid spaces in the INL and OPL, hyperreflective foci in the ONL of the retina, degeneration and atrophy in the PL and pool of extracellular fluid at the RPE of the retina.
